# The characteristics and clinical significance of atypical mitosis in breast cancer

**DOI:** 10.1038/s41379-022-01080-0

**Published:** 2022-05-02

**Authors:** Ayat Lashen, Michael S. Toss, Mansour Alsaleem, Andrew R Green, Nigel P. Mongan, Emad Rakha

**Affiliations:** 1 Academic Unit for Translational Medical Sciences, School of Medicine, University of Nottingham, Nottingham, UK; 2 Department of Pathology, Faculty of Medicine, Menoufia University, Shebin El Kom, Egypt; 3 Department of Applied Medical Science, Applied College, Qassim University, Qassim, Saudi Arabia; 4 Nottingham Breast Cancer Research Centre, University of Nottingham, Nottingham, UK; 5 School of Veterinary Medicine and Sciences, University of Nottingham, Nottingham, UK; 6 Department of Pharmacology, Weill Cornell Medicine, New York, NY USA

**Keywords:** Mitosis, Breast cancer

## Abstract

Atypical mitosis is considered a feature of malignancy, however, its significance in breast cancer (BC) remains elusive. Here, we aimed to assess the clinical value of atypical mitoses in BC and to explore their underlying molecular features. Atypical and typical mitotic figures were quantified and correlated with clinicopathological variables in a large cohort of primary BC tissue sections (*n* = 846) using digitalized hematoxylin and eosin whole-slide images (WSIs). In addition, atypical mitoses were assessed in The Cancer Genome Atlas (TCGA) BC dataset (*n* = 1032) and were linked to the genetic alterations and pathways. In this study, the median of typical mitoses was 17 per 3 mm^2^ (range 0–120 mitoses), while the median of atypical mitoses was 4 (range 0–103 mitoses). High atypical mitoses were significantly associated with parameters characteristic of aggressive tumor behavior. The total number of mitoses, and a high atypical-to-typical mitoses ratio (>0.27) were associated with poor BC specific survival (BCSS), (*p* = 0.04 and 0.01, respectively). The atypical-to-typical mitoses ratio dichotomized triple negative-BC (TNBC) patients into two distinct groups in terms of the association with the outcome, while the overall number of mitoses was not. Moreover, TNBC patients with high atypical-to-typical mitoses ratio treated with adjuvant chemotherapy were associated with shorter survival (*p* = 0.003). Transcriptomic analysis of the TCGA-BRCA cohort dichotomized based on atypical mitoses identified 2494 differentially expressed genes. These included genes linked to pathways involved in chromosomal localization and segregation, centrosome assembly, spindle and microtubule formation, regulation of cell cycle and DNA repair. To conclude, the atypical-to-typical mitoses ratio has prognostic value independent of the overall mitotic count in BC patients and could predict the response to chemotherapy in TNBC.

## Introduction

The proliferative activity of breast cancer (BC) can be considered a surrogate indicator of tumor aggressiveness and provides an important prognostic factor linked to the response to chemotherapy^1–3^. Visual quantification of mitotic figures in hematoxylin and eosin (H&E) stained BC histological sections, defined as the mitotic score, is the gold standard assessment method that reflects the proliferative status of BC^4,5^. In addition, it is an integral component of the Nottingham Grading System^6^.

Mitotic figures are either typical or atypical^7,8^. Atypical mitosis refers to presence of unusual, dysregulated, and random assembly of nuclear materials within the dividing cells which results in abnormal mitotic morphology which also reflects underlying genomic abnormalities such as chromosomal instability, telomere dysfunction, and aneuploidy^9,10^. The presence of atypical mitotic figures is generally acknowledged to be a feature of malignancy and provides prognostic value in certain tumors such as urothelial^11^ and pancreatic^12^ carcinomas. Although some studies utilizing a limited number of cases have reported an association between atypical mitoses and poor outcome in BC^9^, validation studies and characterization of atypical mitoses score in BC remain lacking.

In this study, we hypothesized that atypical mitoses provide additional prognostic significance in BC. We have visually quantified atypical mitoses in a large cohort of BC using digitalized whole slide images (WSIs) to assess the relationship between atypical mitoses and patient outcomes. We also used the publicly available RNA sequencing data (RNA-Seq) from The Cancer Genome Atlas (TCGA) BC dataset^13,14^ to relate atypical mitoses to the underlying molecular changes and pathways.

## Materials and methods

### Study cohort

A total of 846 primary early-stage BC were included. Detailed clinicopathological data including tumor size, histological tumor grade, histologic type, lymph node status, lymphovascular invasion (LVI), Nottingham Prognostic Index (NPI) and molecular subtypes^15^ was available (Supplementary Table 1). Molecular subtypes were determined based on estrogen receptor (ER), progesterone receptor (PR) and the human epidermal growth factor 2 (HER2) into luminal, triple negative (TN) and HER2 enriched BC sub-classes as previously described^16^. All samples were fixed and processed using a standardized method according to the existing protocols^17^. This BC cohort received uniform adjuvant treatment based on NPI and ER status. None of the patients included in the study received neoadjuvant therapy. The median follow-up time was 138 months with available outcome data, including BC‐specific survival (BCSS) defined as the time (in months) from the date of the primary surgery to the time of death from BC, and distant metastasis‐free survival (DMFS) defined as the time (in months) from the primary surgery until the first event of distant metastasis.

### Tissue preparation and digitalization

In the Nottingham series of cases, we have reviewed 3–4 tumor sections per case, and the representative section, defined as the section with highest tumor grade and burden, was selected to be included in the study. Freshly cut, 4‐μm full‐face H&E sections were prepared from the selected formalin-fixed paraffin-embedded (FFPE) blocks and digitally scanned at 40× magnification using a high‐throughput automated scanner (Panoramic 250 Flash III: 3D‐Histech, Budapest, Hungary). WSIs were viewed by Case Viewer software (version 2.2.0.85; 3D‐Histech) on a full‐screen panel (21-inch screen with 1366 × 768 pixels resolution). Regarding the TCGA cohort, the majority of cases had a single online available H&E digitalized image prepared from FFPE tissue blocks. Few cases had two or more WSIs for the invasive tumor, and the representative section, defined as described above, was selected to be included in this study.

### Mitoses identification and counting

**Fig. 1:**
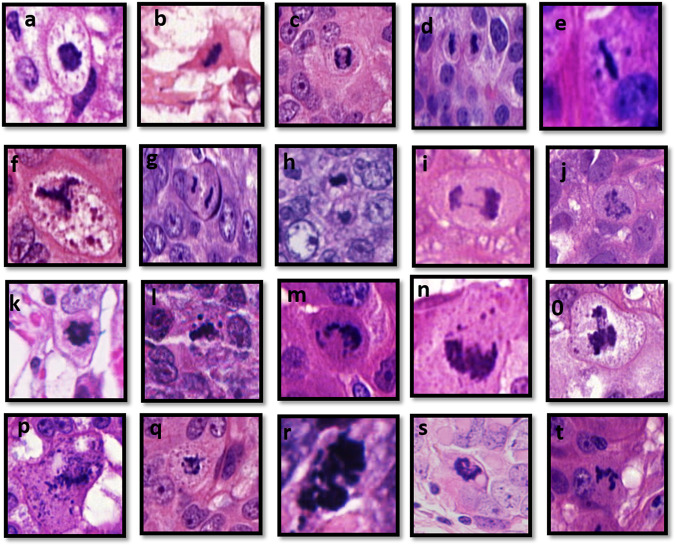
Features of mitosis. Morphological features of typical and atypical mitosis (40×); **a** a prophase shows round hyperchromatic structure with hairy-like appearance, **b** metaphase shows curvilinear structure with slightly eosinophilic cytoplasm, **c** anaphase shows 2 pulled away uniform chromatin masses, **d** telophase shows 2 uniform completely separate cells; **e** lag atypical mitosis shows 2 unattached chromosomes at each side of metaphase plate; **f** tripolar atypical mitosis shows three poles with hairy outline and slightly granular eosinophilic cytoplasm; **g** polar asymmetry shows either 2 pulled- away chromatid unequal in size, enlarged size and slightly eosinophilic cytoplasm or **h** two non-uniform separated cells; **i** anaphase bridge shows a string of chromatin extending from one pole of the anaphase to the other connected poles; **j** dispersed mitosis shows non-clumped multiple chromosomes with slightly eosinophilic cytoplasm and enlarged in size; **k** ring atypical mitosis shows ring-like structure with hairy outline; **l** lag atypical mitosis shows one un-attached chromosomes at one side of metaphase plate; **m**–**t** other forms of atypical mitoses showing asymmetry of the chromatin masses within the cells.

The overall number of mitoses was evaluated in hotspots within an area of 3 mm^2^ on digitalized WSI, which is equivalent to 10 high-power fields (HPFs) of the wide field microscope diameter used in routine practice as per our previous study^18^. Hotspot areas were selected based on being the most cellular area and was usually located at the peripheral invasive front of the tumor^7,19^. Initial examination of digital images was carried out at 10× magnification to identify the cellular regions harboring the highest mitotic counts which were quantified at 40× magnification. Areas of fibrosis or necrosis were excluded. Ambiguous structures such as cells with dark hyperchromatic nuclei and/or apoptotic cells were excluded^8^. Quantifying typical and atypical mitoses was performed separately as the following:

(A) Typical mitoses were identified by their morphology, in addition to the common features of absence of the nuclear membrane and clearly visible hairy extension of nuclear materials, typical mitosis is characterized by specific arrangement of the chromatin. This includes either clotted (prophase), in plane (metaphase/anaphase), or in separate clots (telophase)^8,20^.
(B) Atypical mitoses were defined as mitoses with any morphological appearance other than the typical forms^21^. These include (i) lag atypical mitosis defined as a rod-like structure with one or more unattached chromosome at one side or both sides of metaphase; (ii) spindle multipolarity which shows three attached poles (tripolar), four attached poles (tetrapolar) or more than four poles (multipolar); (iii) dispersed mitosis which is represented by multiple dispersed chromosomes with no specific shapes; (iv) polar asymmetry in which there are two separated masses with unequal size; (v) anaphase bridge that is characterized with a string of chromosome attached to one poles of anaphase; (vi) ring mitosis which is an unusual feature in which chromosomes are displaced to the periphery of the cell^8^ (Fig. 1). Atypical-to-typical mitoses ratio was calculated by the number of atypical mitoses (AM) divided by number of the typical mitoses (TM) per 3 mm^2^ (=AM/AT/3 mm^2^). Mitotic scores were analyzed with respect to detailed clinicopathological parameters and outcome data. Evaluation of all mitoses was carried out by one observer (AL), who is a well-trained pathologist with more than 5 years’ experience in histopathology and was supervised by a specialized breast pathology consultant (ER). In addition, and to assess the inter-observer concordance, another trained pathologist (MT) scored mitoses in 20% of the study cohort and the agreement between both observers was excellent (kappa = 0.85).

### Deciphering the potential molecular changes linked with atypical mitoses

TCGA-BRCA cohort (*n* = 1032) (cBioPortal.org)^22^ was used to study the potential underlying molecular alterations and the differentially expressed genes (DEGs) linked to atypical mitoses. In this cohort, candidate BC histological evaluation was achieved by consensus of a pathology committee^13^. RNA sequencing was carried out with the Illumina HiSeq platform, and the data was processed using previously described methods^23^. The resulting sequencing reads were aligned to the human hg19 genome assembly using MapSlice^24^. RSEM was used to quantify gene expression for the transcript models corresponding to the TCGA GAF 2.13 and was normalized within samples to a fixed upper quartile. Atypical and typical mitoses were counted at 40× magnification per 3 mm^2^ using the same methodology as described above. DEGs linked with atypical and typical mitoses were identified using the DESeq2 R statistical tool^25^, used for differential analysis of count data, using shrinkage estimation for dispersions and fold changes to improve stability and interpretability of estimates. Significant differentially expressed genes were identified based on log2 fold change (≥±1) combined with adjusted *p* value (<0.05). The web-based gene set enrichment analysis tool (WebGestalt) was used to explore significantly enriched pathways and gene ontologies (GO) based on the identified DEGs^26^. The common DEGs were identified between atypical and typical mitoses using the Venny version 2.0 tool^27^.

### Statistical analysis

Statistical analysis was carried out using SPSS v.26 (Chicago, IL, USA). For statistical analysis purposes, atypical mitoses, the overall mitotic counts, and atypical-to-typical mitoses ratio were categorized based on the median, into low and high groups, and were correlated with the clinicopathologic parameters using Chi-square test. The degree of inter-observer agreement in atypical mitoses scoring was assessed statistically using Cohen’s Kappa test. Logistic regression analysis was used to assess the effect of other confounders in the association of atypical mitoses with other variables. The Kaplan–Meier survival curves and log-rank test were used for outcome associations in the whole cohort and in various molecular subtypes. For all tests, *P* < 0.05 (two‐tailed) were considered statistically significant.

## Results

### Distribution of the overall and atypical mitoses

The median number of the overall mitoses (both typical and atypical forms combined) was 21 (range 0–153 mitoses per 3 mm^2^). The median of typical mitoses was 17 (range 0–120 mitoses), while the median of atypical mitoses was 4 (range 0–103 mitoses). One or more atypical mitoses were present in 77% of the cases (Supplementary Fig. 1).

It was noted that the distribution of typical and atypical mitoses was not uniform between tumors and some cases show high overall mitotic count but with a low number of atypical mitoses while others showed high proportion of atypical mitoses compared to the overall number of mitoses. Therefore, it was hypothesized that the ratio between atypical and typical mitoses could be prognostically informative. For this reason, the atypical-to-typical mitoses ratio was calculated in each case. The mean of atypical-to-typical mitoses ratio was 0.4 (median = 0.27; range 0–4).

### Correlation with other clinicopathological parameters

There was a significant association between high atypical mitoses and high grade, larger tumor size, NST tumor type, the poor prognostic NPI group and TNBC phenotype (Table 1). A significant association was confirmed between high overall mitotic count (>21 mitoses per 3 mm^2^) and other parameters characteristic of aggressive tumor behavior including high tumor grade, larger tumor size, NST histological type, the moderate and poor prognostic NPI groups and (TNBC) phenotype (Supplementary Table 2). High atypical-to-typical mitoses ratio showed similar associations with clinicopathological parameters (Table 1). Logistic regression analysis showed that overall mitoses and atypical mitoses are independently associated with tumor size and histologic tumor type regardless of the degree of tubule formation and pleomorphism score.

**Table 1:** Relationship between atypical mitoses, atypical-to-typical mitoses ratio and clinicopathological parameters.

Categories	Number (%)	Low atypical mitoses ≤ 4	High atypical mitoses > 4	X^2^ *P* value	Low ratio ≤ 0.27	High ratio > 0.27	X^2^ *P* value
Tumor size							
≤2 cm	487 (58%)	289 (59%)	198 (41%)	22.4	267 (55%)	220 (45%)	10.2
>2 cm	359 (42%)	154 (43%)	205 (57)	**<0.00 01**	157 (44%)	202 (56)	**<0.00 01**
Tumor grade							
Grade 1	75 (9%)	73 (97%)	2 (3%)	229.3	66 (88%)	9 (12%)	126.3
Grade2	191 (23%)	168 (88%)	23 (12%)	**<0.0001**	142 (74%)	49 (26%)	**<0.0001**
Grade 3	580 (68%)	202 (35%)	378 (65%)		216 (37%)	364 (63%)	
Histologic types							
No special type (NST)	639 (75%)	268 (42%)	371 (58%)	115.4	269 (42%)	371 (58%)	71.0
Lobular	53 (6%)	44 (83%)	9 (17%)	**<0.00 01**	34 (64%)	19 (36%)	**<0.00 01**
Other special types	116 (14%)	102 (88%)	14 (12%)		93 (80%)	23 (20%)	
Mixed NST	38 (5%)	29 (76%)	9 (24%)		28 (74%)	10 (26%)	
Molecular subtype							
Luminal	380 (46%)	293 (77%)	87 (23%)	117.5	260 (68%)	120 (32%)	93.4
Triple negative	324 (40%)	92 (28%)	232 (72%)	**<0.0001**	108 (33%)	216 (67%)	**<0.0001**
HER2+	116 (14%)	44 (38%)	72 (62%)		45 (47%)	71 (53%)	
Lymph node status							
Negative	533 (63%)	285 (54%)	248 (46%)	0.6	273 (51%)	260 (49%)	0.6
Positive	312 (37%)	158 (51%)	154 (49%)	0.43	151 (48%)	161 (52%)	0.4
Lymphovascular invasion							
Absent	576 (68%)	305 (53%)	271 (47%)	0.3	304 (53%)	272 (47%)	5.1
Present	270 (32%)	138 (51%)	132 (49%)	0.6	120 (44%)	150 (56%)	**0.02**
Nottingham Prognostic index							
Good prognostic group	160 (19%)	149 (93%)	11 (7%)	132.3	133 (83%)	27 (17%)	86.1
Moderate prognostic group	524 (62%)	231 (44%)	293 (56%)	**<0.0001**	226 (43%)	298 (57%)	**<0.0001**
Poor prognostic group	161 (19%)	63 (39%)	98 (61%)		65 (40%)	96 (60%)	

Significant *P* values are in bold.

*HER2* human epidermal growth factor receptor 2, *ratio* atypical-to-typical mitoses ratio.

### Outcome analysis

**Fig. 2:**
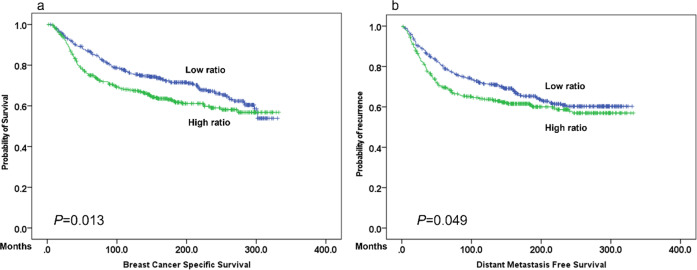
Association of atypical-to-typical mitosis ratio with outcome. Kaplan–plots showing associations of atypical-to-typical mitoses ratio with **a** Breast Cancer Specific Survival (BCSS) and with **b** Distant Metastasis Free Survival (DMFS).

A significant association was confirmed between the overall mitoses score, but not atypical mitoses, and patient outcome in terms of shorter BCSS (*p* = 0.04) and DMFS (*p* = 0.03). However, when the ratio was considered, high atypical-to-typical mitotic ratio showed a strong association with poor outcome (*p* = 0.013) (Fig. 2).

**Fig. 3:**
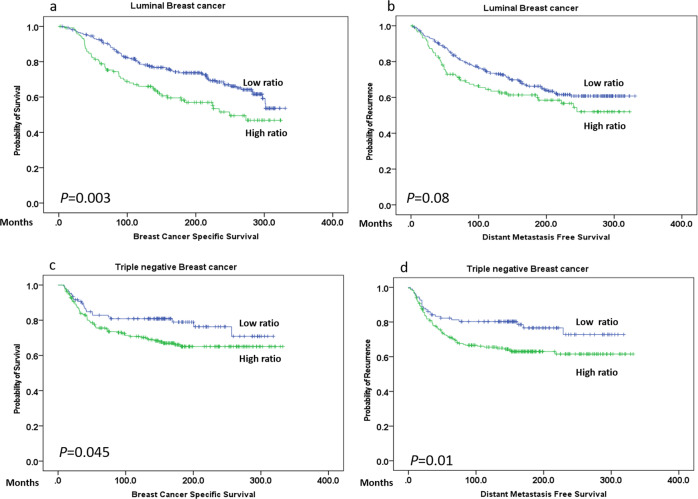
Association of atypical-to- typical mitosis ratio with outcome in different molecular classes. **a**, **b** Kaplan–Meier plots showing associations of atypical-to-typical mitoses ratio with BCSS in luminal while **c**, **d** association of high ratio with both poor BCSS and DMFS in triple negative breast cancer subtype.

**Fig. 4:**
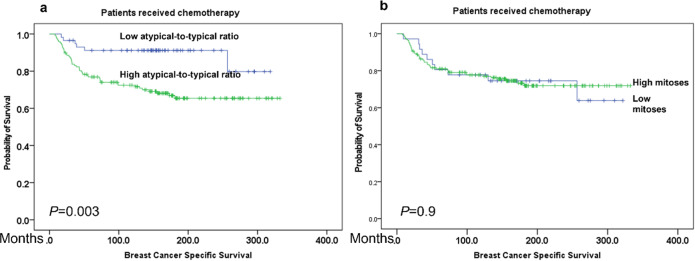
Association of atypical-to- typical mitosis ratio with chemotherapy response. Kaplan–Meier analysis showing **a** significant association between BCSS and atypical-to-typical mitoses ratio in TNBC patients who received chemotherapy while there was no association between overall mitoses and BCSS in those patients (**b**).

When the molecular classes were considered, the overall mitoses score showed an association with shorter BCSS in luminal BC (*P* = 0.001) but not in either TNBC nor in the HER2 subtypes. Similarly, a significant association between high atypical mitoses and both shorter BCSS and DMFS was observed in the luminal BC subtype (*p* values = 0.006 and 0.03, respectively) (Supplementary Fig. 2) but not in the TNBC, nor the HER2 class. However, when the atypical-to-typical mitoses ratio was applied, a significant association with outcome was observed not only in luminal BC (*p* = 0.003), but also in TNBC subtype (*p* = 0.01) (Fig. 3). When the whole cohort was stratified based on the adjuvant chemotherapy treatment, there were significant negative associations between overall mitoses, and the atypical-to-typical mitoses ratio and outcome (*p* = 0.001 and 0.028, respectively) in the chemotherapy naïve group but not in chemotherapy treated patients (*p* = 0.9 and 0.2, respectively). However, when the analysis was carried out on various molecular subtypes, high atypical-to-typical mitoses ratio, showed shorter BCSS in TNBC patients who received chemotherapy (*p* = 0.003), while this association was not observed when the overall mitoses was considered (*p* = 0.9) (Fig. 4).

### Atypical mitoses and the transcriptomic profiling

In the TCGA-BRCA cohort, the median of overall mitoses was 18 (range 0–148 mitoses per case) while the median of atypical mitoses was 2 (range 0–97 mitoses). The median of typical mitoses was 13 (range 0–116) and the mean atypical-to-typical mitoses ratio was 0.3 (median = 0.2; range 0 to 5). There was statistically significant difference in the mitoses between the Nottingham and TCGA cohort (*P* = 0.001). Similar to the previous finding, high atypical mitoses group was significantly associated with parameters characteristic of aggressive behavior like larger tumor size, high tumor grade and TNBC. Similar observation was confirmed regarding high overall mitoses and high ratio.

**Fig. 5:**
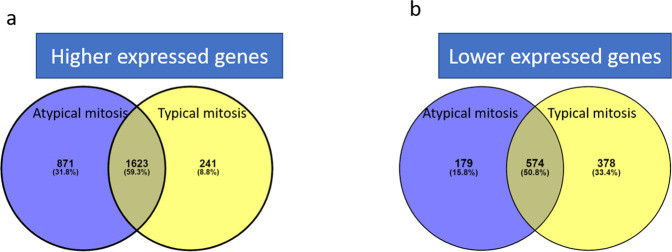
Differential expression genes. Venn diagram of obtained Differentially expressed genes (DEG) shows **a** the overlap between the higher expressed genes associated with atypical and typical mitoses while **b**) the overlap between the lower expressed genes (DEGs) associated with atypical and typical mitoses. Differentially expressed genes were chosen based on fold change (≥±1) combined with False discovery rate (FDR) < 0.05.

Differential gene expression analysis identified a set of genes (*n* = 3247) that were associated with atypical mitoses, where 2494 genes were significantly associated with high atypical mitoses and 753 genes were associated with low score cases. Regarding typical mitoses, 1864 genes were significantly associated with high typical mitoses whereas 952 genes were associated with low typical mitoses (Supplementary Fig. 3). There were about 60% (1623) genes commonly up-regulated across atypical and typical mitoses and 574 genes were commonly down-regulated) (Fig. 5). The genes that showed higher expression in cases with high atypical mitoses were involved in specific biological pathways including, regulation of chromosomal organization, localization, and segregation, double strand DNA breakdown repair, regulation of cell cycle, and cell cycle check points as shown in (Table 2).

**Table 2:** The Biological process category by the Gene Ontology shows the common biological processes that are significantly associated with high atypical mitoses.

Gene Set	Description	Size/ overlap	Adjusted *P* value	Gene symbol
GO:0033044	Regulation of chromosome organization	18/18	0.0001	HIST1H1A- PADI2- AURKB- CDC20- TTK- PLK1
GO:0006302	Double-strand break repair	9/8	0.001	TRIP13- CDC45- CDCA5- EXO1 RAD54L- RPA4- AUNIP
GO:0048285	Organelle fission	41/32	0.004	UBE2C-TTK-KIF2C- ASZ1- CEP55 TPX2- CDCA8
GO:0050000	Chromosome localization	9/9	0.004	KIF2C- CEP55-FAM83D- NDC80 CDCA8- KIF14-TERB2
GO:0007059	Chromosome segregation	30/25	0.003	AURKB- CDC20- BIRC5- SKA1 TTK- FAM83D- KIF2C-PLK1
GO:0045930	Regulation of cell cycle	14/13	0.01	AURKB- CDC20-TTK-TICRR STK33- AURKA- NDC80

For the Gene Set Enrichment Analysis (GSEA) method, the category size is determined by the number of annotated genes in the category and the reference gene list. The category overlap is calculated by the overlapping differentially expressed genes (fold change (≥±1) combined with adjusted *p* value (<0.05) found in our differential gene expression analysis and those found in the gene list. The category *p* value represents the weighted set cover and maximum coverage known as size-constrained weighted set cover, in which weights are assigned to gene sets with lower enrichment *p* values.

## Discussion

Atypical mitoses are characterized by abnormalities in the mitotic spindle symmetry, abnormal sister chromatid separation and are thought to reflect genetic abnormalities that underlie malignant phenotypes^12^ and aggressive behavior^9,21,28^. However, detailed characterization of atypical mitoses in BC remained to be defined. In the present study, the prognostic and molecular significance of atypical mitoses in BC utilizing two large well characterized cohorts was evaluated. We hypothesized that atypical mitoses, which can be easily assessed in routine histological specimens provide prognostic information similar to or better than other costly morphological and molecular prognostic parameters^9^.

In a preliminary study of BC, we found that atypical mitoses are sparse in BC with low overall mitotic score^20^, so in the current study, we sought to enrich our local cohort with cases rich in mitotic figures including BC cases that show mitotic grading scores 2 and 3. In the TCGA cohort, the whole cohort was assessed; therefore, the median number of overall mitoses was higher in the Nottingham cohort. Also, to avoid comparing BC with high atypical mitoses against BC with low overall mitoses scores that may bias the results as the findings may represent the mitotic activity rather than the atypicality of mitosis, we analyzed not only atypical mitoses but also the atypical-to-typical mitoses ratio. In this study, although atypical mitosis and overall mitosis showed strong associations with the histologic grade, which is expected as mitosis score is one of the grade components, our results showed that atypical mitoses had independent association with tumor size and histologic tumor type regardless the degree of tubule formation and pleomorphism score of the tumor. This highlights the clinical and biological importance of identification of atypical mitoses in BC.

Our results showed strong associations between high atypical mitoses score and other various parameters characteristic of aggressive tumor behavior and indicate that not only the overall mitotic score but also the atypical mitoses score and most importantly in the context of this study, the atypical-to-typical mitoses ratio, provide important prognostic value. Here we show a cut-off point of 4 segregated atypical mitoses into low and high groups, however pathologists may be reluctant to use only 4 atypical mitoses for prognostic stratification of BC as in routine practice cut-off point of 8–12 is used to differentiate score 1 from 2 overall mitosis based on the currently used microscopes. For this reason, the atypical-to-typical mitoses ratio may be more practical, pragmatic, and reflective of the intrinsic proliferative and genomic molecular phenotype of the tumor.

In the whole BC cohort, atypical mitoses per se did not show association with outcome though, when the cohort was stratified into molecular sub-classes, atypical mitoses segregated luminal BC subtypes into two distinct prognostic groups. Indeed, a previous study, albeit conducted on a small cohort with limited clinical information, showed that detection of atypical mitoses is a poor prognostic indicator in BC^9^. Importantly, when the atypical-to-typical mitoses ratio was considered, prognostic stratification was identified not only in the whole cohort or in the luminal BC class but also in the TNBC subtype. The overall mitoses were not prognostic in TNBC neither as scores nor as a continuous variable. This may reflect the high proliferative activity of TNBC and that the atypical-to-typical mitoses ratio reflects the combination of the proliferative activity and the genomic alterations of these tumors.

When the cohort was stratified based on adjuvant chemotherapy, there was an association between shorter survival and BC with high overall mitoses or high atypical-to-typical mitoses ratio in the chemotherapy naïve group. This association disappeared in the chemotherapy treated patients. This is likely to reflect the better response of BC with high proliferative activity to chemotherapy resulting in outcome similar to those with low proliferation. When BC was classified based the molecular classes, our results showed a significant improvement in the outcome of TNBC patients with low atypical-to-typical mitoses ratio when they are treated with chemotherapy compared to those with high ratio. In TNBC patients who did not receive chemotherapy, the outcome of those with low atypical-to-typical mitoses ratio was not different to that in BC patients with the high ratio. When chemotherapy was given, patients with low ratio responded well and showed significantly longer survival compared to those with high ratio. Such observation was not identified when the overall mitoses was considered in this molecular class of BC patients. These findings suggested that high-risk TNBC patients with high atypical-to-typical mitoses ratio have limited response and they should be offered additional therapy, whenever possible.

Although there remains a lot of attention to the use of the molecular based assays for prognostication in women with BC and this is likely to continue at least for some time, there are factors need to be considered. The current study provided a morphological feature (atypical-to-typical mitoses ratio) that can be combined to the other well-known prognostic and predictive factors to add better risk stratification tool in BC as a complementary or even a replacement of the expensive molecular assays. In the era of digital pathology and artificial intelligence, assessment of atypical mitoses and its incorporation within the prognostic and predictive AI-based algorithms is likely to provide a cost-efficient risk assessment tool. Moreover, the actual performance of the available molecular tests is largely similar to the well-established morphological and clinicopathological parameters when assessed accurately using robust methods. Head-to-head comparison of the molecular assays in BC showed similar concordance levels to that reported for histological grade and mitotic scores when assessed in well-fixed samples (Kappa scores = 0.39–0.55), with discordant results being recorded in 41% of patients^29–32^. The current view is that molecular tests are likely to be cost-effective, but an optimal test is yet to be identified^33,34^. In addition, most of the routinely available molecular tests are applied to the indeterminate risk BC group, with limited performance in the TNBC and HER2 positive subtypes, unlike the morphological factors such as the one reported in this study. In addition, due to costs, morphological and clinicopathological variables remains as the main prognostic markers used in the low and middle income countries. Finally, the current evidence indicates that the best approach is to combine molecular tests with the clinicopathological parameters such as the one reported in this study to obtain the best stratification^35^.

In this study, 77% of the differentially expressed genes (DEG) identified were higher in tumors harboring atypical mitoses and were associated with biological pathways and mechanisms linked to chromosomal localization and segregation, centrosome spindle and microtubule, micro-RNA and DNA breakdown. These finding are consistent with other studies linking atypical mitoses association with genomic instability, telomere dysfunction, chromosomal abnormalities, and aneuploidy^36^. Some studies have reported that chromosomal instability and telomere dysfunction are the main contributors to atypical mitoses in malignancy and that atypical mitosis can be considered a morphological marker of chromosomal instability^37,38^. Additionally, It was proposed that presence of atypical mitotic figures is caused by specific centrosomal alterations in each neoplasm, and may be considered as a distinct event that is possibly involved in carcinogenesis^39^. Aneuploidy is one of the common characteristic features of cancer cells and is related to poor clinical outcomes^40^. The underlying mechanism for aneuploidy in cancer may be a defect in the mitotic process that is required to segregate duplicated chromosomes into daughter cells^41^. Atypical mitosis could provide a significant morphological signature of underlying chromosomal instability and aneuploidy of tumor cells.

The main limitation of using the atypical-to-typical mitoses ratio in routine practice, is that it depends on independent assessment of both typical and atypical count separately, which may be considered a time-consuming task for pathologists. However, with the emergence of advanced digital pathology and artificial intelligence/machine learn (AI/ML) algorithms, it should be feasible to develop automated image analysis approaches to accurately distinguish and quantify typical and atypical mitoses. In conclusion, our findings suggest that the BC tumors with atypical mitoses possess molecular features associated with an aggressive phenotype, higher risk of metastasis and chemotherapy resistance. Our study supports further investigation of the use of quantitative assessment of atypical mitoses in the context of overall mitotic number as a prognostic indicator in BC.

## Supplementary information

Supplementary material

## Data Availability

All data used in this study are archived and could be available on a reasonable request.

## References

[1] Rakha, E. A., Reis-Filho, J. S., Baehner, F., Dabbs, D. J., Decker, T., Eusebi, V. et al. Breast cancer prognostic classification in the molecular era: the role of histological grade. *Breast Cancer Res.* **12**, 207 (2010). 10.1186/bcr2607 PMC2949637 20804570

[2] Smith, I., Robertson, J., Kilburn, L., Wilcox, M., Evans, A., Holcombe, C. et al. Long-term outcome and prognostic value of Ki67 after perioperative endocrine therapy in postmenopausal women with hormone-sensitive early breast cancer (POETIC): an open-label, multicentre, parallel-group, randomised, phase 3 trial. *Lancet Oncol.* **21**, 1443–1454 (2020). 10.1016/S1470-2045(20)30458-7 PMC7606901 33152284

[3] Fasching, P. A., Heusinger, K., Haeberle, L., Niklos, M., Hein, A., Bayer, C. M. et al. Ki67, chemotherapy response, and prognosis in breast cancer patients receiving neoadjuvant treatment. *BMC Cancer* **11**, 486 (2011). 10.1186/1471-2407-11-486 PMC3262864 22081974

[4] Beresford, M. J., Wilson, G. D. & Makris, A. Measuring proliferation in breast cancer: practicalities and applications. *Breast Cancer Res*. **8**, 216-216 (2006). 10.1186/bcr1618 PMC1797032 17164010

[5] Aleskandarany, M. A., Green, A. R., Benhasouna, A. A., Barros, F. F., Neal, K., Reis-Filho, J. S. et al. Prognostic value of proliferation assay in the luminal, HER2-positive, and triple-negative biologic classes of breast cancer. *Breast Cancer Res.* **14**, R3 (2012). 10.1186/bcr3084 PMC3496118 22225836

[6] van Dooijeweert, C., van Diest, P. J. & Ellis, I. O. Grading of invasive breast carcinoma: the way forward. *Virchows Archiv*10.1007/s00428-021-03141-2 (2021). 10.1007/s00428-021-03141-2 PMC8983621 34196797

[7] Al-Janabi, S., van Slooten, H. J., Visser, M., van der Ploeg, T., van Diest, P. J. & Jiwa, M. Evaluation of mitotic activity index in breast cancer using whole slide digital images. *PLoS One* **8**, e82576 (2013). 10.1371/journal.pone.0082576 PMC3875418 24386102

[8] Ibrahim, A., Lashen, A., Toss, M., Mihai, R. & Rakha, E. Assessment of mitotic activity in breast cancer: revisited in the digital pathology era. *J. Clin. Pathol.*10.1136/jclinpath-2021-207742 (2021). 10.1136/jclinpath-2021-207742 34556501

[9] Ohashi, R., Namimatsu, S., Sakatani, T., Naito, Z., Takei, H. & Shimizu, A. Prognostic utility of atypical mitoses in patients with breast cancer: a comparative study with Ki67 and phosphohistone H3. *J. Surg. Oncol.* **118**, 557–567 (2018). 10.1002/jso.25152 30098295

[10] Shekhar, M. P., Lyakhovich, A., Visscher, D. W., Heng, H. & Kondrat, N. Rad6 overexpression induces multinucleation, centrosome amplification, abnormal mitosis, aneuploidy, and transformation. *Cancer Res.* **62**, 2115–2124 (2002). 11929833

[11] Jin, Y., Stewénius, Y., Lindgren, D., Frigyesi, A., Calcagnile, O., Jonson, T. et al. Distinct mitotic segregation errors mediate chromosomal instability in aggressive urothelial cancers. *Clin. Cancer Res.* **13**, 1703 (2007). 10.1158/1078-0432.CCR-06-2705 17363523

[12] Matsuda, Y., Yoshimura, H., Ishiwata, T., Sumiyoshi, H., Matsushita, A., Nakamura, Y. et al. Mitotic index and multipolar mitosis in routine histologic sections as prognostic markers of pancreatic cancers: a clinicopathological study. *Pancreatology* **16**, 127–132 (2016). 10.1016/j.pan.2015.10.005 26585687

[13] Koboldt, D. C., Fulton, R. S., McLellan, M. D., Schmidt, H., Kalicki-Veizer, J., McMichael, J. F. et al. Comprehensive molecular portraits of human breast tumours. *Nature* **490**, 61–70 (2012). 10.1038/nature11412 PMC3465532 23000897

[14] Weinstein, J. N., Collisson, E. A., Mills, G. B., Shaw, K. R., Ozenberger, B. A., Ellrott, K. et al. The Cancer Genome Atlas Pan-Cancer analysis project. *Nat. Genet* **45**, 1113–1120 (2013). 10.1038/ng.2764 PMC3919969 24071849

[15] Galea, M. H., Blamey, R. W., Elston, C. E. & Ellis, I. O. The Nottingham Prognostic Index in primary breast cancer. *Breast Cancer Res. Treat* **22**, 207–219 (1992). 10.1007/BF01840834 1391987

[16] Rakha, E. A., Agarwal, D., Green, A. R., Ashankyty, I., Ellis, I. O., Ball, G. et al. Prognostic stratification of oestrogen receptor-positive HER2-negative lymph node-negative class of breast cancer. *Histopathology* **70**, 622–631 (2017). 10.1111/his.13108 27782306

[17] Rakha, E. A., Pinder, S. E., Bartlett, J. M., Ibrahim, M., Starczynski, J., Carder, P. J. et al. Updated UK recommendations for HER2 assessment in breast cancer. *J. Clin. Pathol.* **68**, 93–99 (2015). 10.1136/jclinpath-2014-202571 PMC4316916 25488926

[18] Ibrahim, A., Lashen, A. G., Katayama, A., Mihai, R., Ball, G., Toss, M. S. et al. Defining the area of mitoses counting in invasive breast cancer using whole slide image. *Mod. Pathol.*10.1038/s41379-021-00981-w (2021). 10.1038/s41379-021-00981-w PMC9174050 34897279

[19] Lashen, A. G., Toss, M. S., Katayama, A., Gogna, R., Mongan, N. P. & Rakha, E. A. Assessment of proliferation in breast cancer: cell cycle or mitosis? An observational study. *Histopathology* **79**, 1087–1098 (2021). 10.1111/his.14542 34455622

[20] Lashen, A., Ibrahim, A., Katayama, A., Ball, G., Mihai, R., Toss, M. et al. Visual assessment of mitotic figures in breast cancer: a comparative study between light microscopy and whole slide images. *Histopathology* **79**, 913–925 (2021). 10.1111/his.14543 34455620

[21] Donovan, T. A., Moore, F. M., Bertram, C. A., Luong, R., Bolfa, P., Klopfleisch, R. et al. Mitotic figures-normal, atypical, and imposters: a guide to identification. *Vet. Pathol.* **58**, 243–257 (2021). 10.1177/0300985820980049 33371818

[22] Ciriello, G., Gatza, M. L., Beck, A. H., Wilkerson, M. D., Rhie, S. K., Pastore, A. et al. Comprehensive molecular portraits of invasive lobular breast cancer. *Cell* **163**, 506–519 (2015). 10.1016/j.cell.2015.09.033 PMC4603750 26451490

[23] Hoadley, K. A., Yau, C., Wolf, D. M., Cherniack, A. D., Tamborero, D., Ng, S. et al. Multiplatform analysis of 12 cancer types reveals molecular classification within and across tissues of origin. *Cell* **158**, 929–944 (2014). 10.1016/j.cell.2014.06.049 PMC4152462 25109877

[24] Wang, K., Singh, D., Zeng, Z., Coleman, S. J., Huang, Y., Savich, G. L. et al. MapSplice: Accurate mapping of RNA-seq reads for splice junction discovery. *Nucleic Acids Res.* **38**, e178–e178 (2010). 10.1093/nar/gkq622 PMC2952873 20802226

[25] Love, M. I., Huber, W. & Anders, S. Moderated estimation of fold change and dispersion for RNA-seq data with DESeq2. *Genome Biol.* **15**, 550 (2014). 10.1186/s13059-014-0550-8 PMC4302049 25516281

[26] Zhang, B., Kirov, S. & Snoddy, J. WebGestalt: an integrated system for exploring gene sets in various biological contexts. *Nucleic Acids Res.* **33**, W741-748 (2005). 10.1093/nar/gki475 PMC1160236 15980575

[27] Lin, G., Chai, J., Yuan, S., Mai, C., Cai, L., Murphy, R. W. et al. VennPainter: a tool for the comparison and identification of candidate genes based on Venn diagrams. *PLoS One* **11**, e0154315 (2016). 10.1371/journal.pone.0154315 PMC4847855 27120465

[28] Van Leeuwen, A. M., Pieters, W. J., Hollema, H. & Burger, M. P. Atypical mitotic figures and the mitotic index in cervical intraepithelial neoplasia. *Virchows Arch* **427**, 139–144 (1995). 10.1007/BF00196518 7582243

[29] Natrajan, R. & Weigelt, B. Risk stratification and intrinsic subtype classification of breast cancer: a multi-parameter test to rule them all? *J. Natl. Cancer Inst.* **108**, djw118 (2016). 10.1093/jnci/djw118 27130931

[30] Bartlett, J. M., Bayani, J., Marshall, A., Dunn, J. A., Campbell, A., Cunningham, C. et al. Comparing breast cancer multiparameter tests in the OPTIMA prelim trial: no test is more equal than the others. *J. Natl. Cancer Inst.* **108**, djw050 (2016). 10.1093/jnci/djw050 PMC5939629 27130929

[31] Varnier, R., Sajous, C., de Talhouet, S., Smentek, C., Péron, J., You, B. et al. Using breast cancer gene expression signatures in clinical practice: unsolved issues, ongoing trials and future perspectives. *Cancers* **13**, 4840 (2021). 10.3390/cancers13194840 PMC8508256 34638325

[32] Singh, K., He, X., Kalife, E. T., Ehdaivand, S., Wang, Y. & Sung, C. J. Relationship of histologic grade and histologic subtype with oncotype Dx recurrence score; retrospective review of 863 breast cancer oncotype Dx results. *Breast Cancer Res. Treat* **168**, 29–34 (2018). 10.1007/s10549-017-4619-4 29230662

[33] Hall, P. S., Smith, A., Hulme, C., Vargas-Palacios, A., Makris, A., Hughes-Davies, L. et al. Value of information analysis of multiparameter tests for chemotherapy in early breast cancer: the OPTIMA Prelim Trial. *Value Health* **20**, 1311–1318 (2017). 10.1016/j.jval.2017.04.021 29241890

[34] Ward, S., Scope, A., Rafia, R., Pandor, A., Harnan, S., Evans, P. et al. Gene expression profiling and expanded immunohistochemistry tests to guide the use of adjuvant chemotherapy in breast cancer management: a systematic review and cost-effectiveness analysis. *Health Technol. Assess.* **17**, 1–302 (2013). 10.3310/hta17440 PMC4780957 24088296

[35] Tschodu, D., Ulm, B., Bendrat, K., Lippoldt, J., Gottheil, P., Käs, J. A. et al. Comparative analysis of molecular signatures reveals a hybrid approach in breast cancer: combining the Nottingham Prognostic Index with gene expressions into a hybrid signature. *PLoS One* **17**, e0261035 (2022). 10.1371/journal.pone.0261035 PMC8830616 35143511

[36] Takubo, K., Aida, J., Izumiyama-Shimomura, N., Ishikawa, N., Sawabe, M., Kurabayashi, R. et al. Changes of telomere length with aging. Geriatr. Gerontol. Int. **10**, S197–206 (2010). 10.1111/j.1447-0594.2010.00605.x 20590834

[37] Takubo, K., Fujita, M., Izumiyama, N., Nakamura, K., Ishikawa, N., Poon, S. S. et al. Q-FISH analysis of telomere and chromosome instability in the oesophagus with and without squamous cell carcinoma in situ. *J. Pathol.* **221**, 201–209 (2010). 10.1002/path.2704 20455255

[38] Montgomery, E., Wilentz, R. E., Argani, P., Fisher, C., Hruban, R. H., Kern, S. E. et al. Analysis of anaphase figures in routine histologic sections distinguishes chromosomally unstable from chromosomally stable malignancies. *Cancer Biol. Ther.* **2**, 248–252 (2003). 10.4161/cbt.2.3.362 12878857

[39] Batistatou, A. Mitoses and cancer. *Med. Hypotheses* **63**, 281–282 (2004). 10.1016/j.mehy.2004.02.049 15236791

[40] Hayward, D. G., Clarke, R. B., Faragher, A. J., Pillai, M. R., Hagan, I. M. & Fry, A. M. The centrosomal kinase Nek2 displays elevated levels of protein expression in human breast cancer. *Cancer Res.* **64**, 7370–7376 (2004). 10.1158/0008-5472.CAN-04-0960 15492258

[41] Pihan, G. A., Purohit, A., Wallace, J., Knecht, H., Woda, B., Quesenberry, P. et al. Centrosome defects and genetic instability in malignant tumors. *Cancer Res.* **58**, 3974–3985 (1998). 9731511

