# Supplementary material for: The characteristics and clinical significance of atypical mitosis in breast cancer
Source: Mod Pathol. 2022 May 2;35(10):1341–8. doi: 10.1038/s41379-022-01080-0 (PMC9514994; doi:10.1038/s41379-022-01080-0)
Supplement: Supplementary file 1 — Supplementary material [file 41379_2022_1080_MOESM1_ESM.docx]

**Supplementary materials**

**Supplementary table (1)** **Clinicopathological characteristics of the study cohort**

| Variables | No (%) |
| --- | --- |
| Tumour size  ≤ 2cm  > 2cm | 487 (57.6%)  359 (42.4%) |
| Histologic tumour grade  Grade 1  Grade 2  Grade 3 | 75 (8.8%)  191 (22.6%)  580 (68.6%) |
| Histologic tumour types  No special type (NST)  Lobular  Other special types  NST mixed | 639 (75.5%)  53 (6.2%)  116 (13.7%)  38 (4.6%) |
| Molecular subtypes  Luminal  Triple negative  HER2 + | 380 (44.9%)  324 (83.3%)  116 (13.8%) |
| Lymph node invasion  Absent  Present | 533 (63.1%)  312 (36.9%) |
| Lymhovascular invasion  Absent  Present | 576 (68.1%)  270 (31.9%) |
| Nottingham prognostic index  Good prognostic group  Moderate prognostic group  Poor prognostic group | 160 (18.9%)  524 (62.0%)  161 (19.1%) |

HER2, human epidermal growth factor receptor 2

**Supplementary table (2) Relationship between overall mitoses and clinicopathological parameters**

| **Parameters** | **Low mitoses≤ 21** | **High mitoses>21** | **X^2^**  ***P*-value** | |
| --- | --- | --- | --- | --- |
| **Tumour size**  ≤ 2cm  > 2cm | 284(58%)  143(40%) | 203(42%)  216(60%) | 28. 2 **<0.0001** |  |
| **Tumour grade**  Grade 1  Grade2  Grade 3 | 75 (99%)  172 (91%)  180 (31%) | 1 (1%)  18 (9%)  400 (69%) | 280.3  **<0.0001** |  |
| **Histologic types**  NST  Lobular  Other special types  Mixed tumours | 251(39%)  35(83%)  88(90%)  53(79%) | 388(61%)  7(17%)  10(10%)  14(21%) | 135.5  **<0.0001** |  |
| **Molecular subtype**  Luminal  Triple negative / basal  HER2+ | 298 (78%)  71 (22%)  45 (39%) | 82 (22%)  253(78%)  71 (61%) | 230.8  **<0.0001** |  |
| **Lymph node status**  Negative  Positive | 269 (51%)  158 (51%) | 264 (49%)  154 (49%) | **0.002**  0.91 |  |
| **Lymphovascular invasion**  Absent  Present | 289 (50%)  138 (51%) | 287 (50%)  132 (49%) | 0.1  0.7 |  |
| **Nottingham prognostic index**  Good prognostic group  Moderate prognostic group  Poor prognostic group | 149 (93%)  221 (42%)  57 (35%) | 11 (7%)  303 (58%)  104 (65%) | 145.5  **<0.0001** |  |

HER2, human epidermal growth factor receptor 2 Significant P values are in **bold**

| **No** | **Gene Set** | **Description** | **Size/** **Overlap** | ***P* value** | **Gene symbol** |
| --- | --- | --- | --- | --- | --- |
| **Biological processes** | | | | | |
| 1 | GO:0044772 | Mitotic cell cycle phase transition | 35/27 | 0.02 | MCM10- AURKB- CDC20- UBE2C- FOXM1- TTK- MELK- TICRR- ORC6- CDC25A |
| 2 | GO:0000075 | Cell cycle checkpoint | 16/14 | 0.04 | AURKB - PLK1- ORC1- TRIP13- NDC80- TICRR- TTK |
| 3 | GO:0010721 | negative regulation of cell development | 19/18 | 0.028 | BCL11A-DLL3-EPHA7- GAL-ISL2-KLK8-LHX2-LIN28A-MELTF-MT3 |
| **Cellular processes** | | | | | |
| 1 | GO:0005819 | spindle | 17/16 | 0.001 | AURKB- BIRC5- SKA1- TTK- FAM83D- CDCA8- TPX2- AURKA |
| 2 | GO:0098687 | Chromosomal region | 22/20 | 0.0001 | CENPA- AURKB- BIRC5- SKA1- TTK- KIF2C- PIF1- NDC80- PLK1 |
| 3 | GO:0030496 | Midbody | 8/8 | 0.01 | AURKB- BIRC5- PLK1- CDCA8  ANLN- KIF14 |
| 4 | GO:0045111 | intermediate filament cytoskeleton | 29/18 | 0.0001 | SLC1A6- KRT16- KRT33B- INA- CASP14- S100A8 |

**Supplementary table (3) more biological processes and cellular processes that are significantly associated with high atypical mitosis**

For the Gene Set Enrichment Analysis (GSEA) method, the category size is determined by the number of annotated genes in the category and the reference gene list. The category overlap is calculated by the overlapping differentially expressed genes (fold change (≥±1) combined with adjusted *p*-value (<0.05) found in our differential gene expression analysis and those found in the gene list. The category p value represents the weighted set cover and maximum coverage known as size-constrained weighted set cover, in which weights are assigned to gene sets with lower enrichment p values.


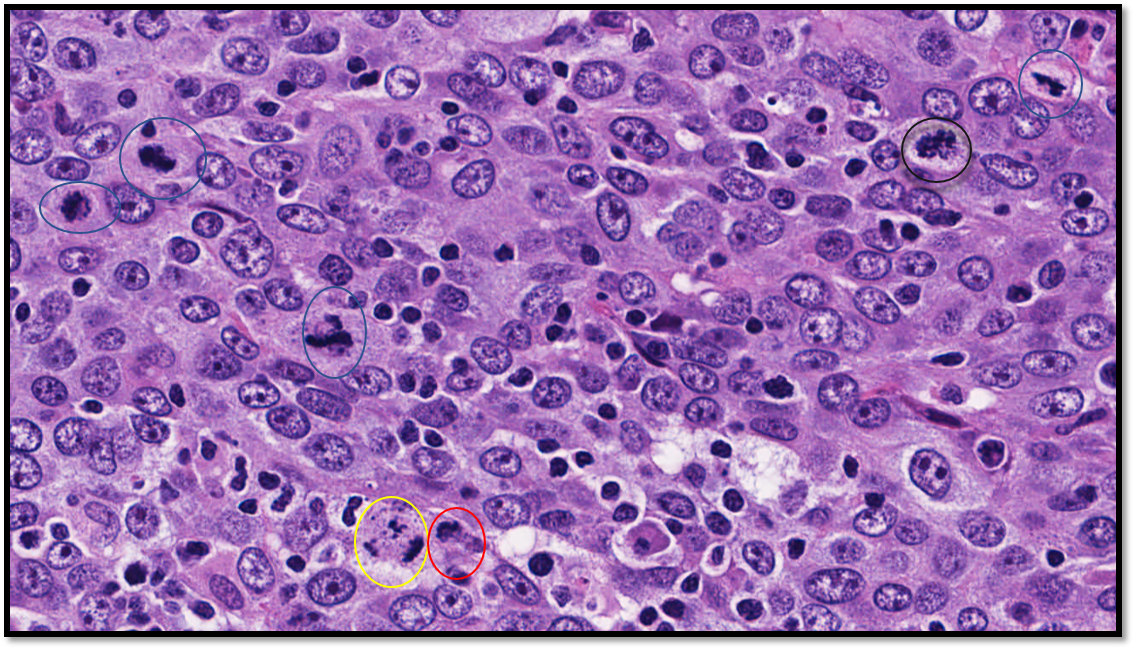


Supplememtary figure (1) A breast cancer case shows multiple atypical mitoses in one high power field (x40): blue circules show different shapes of lag atypical mitoses, yellow circle shows dispersed atypical mitosis, red circle shows polar asymmetry atypical mitosis and the black circle shows other form of atypical mitosis with irregular spikes


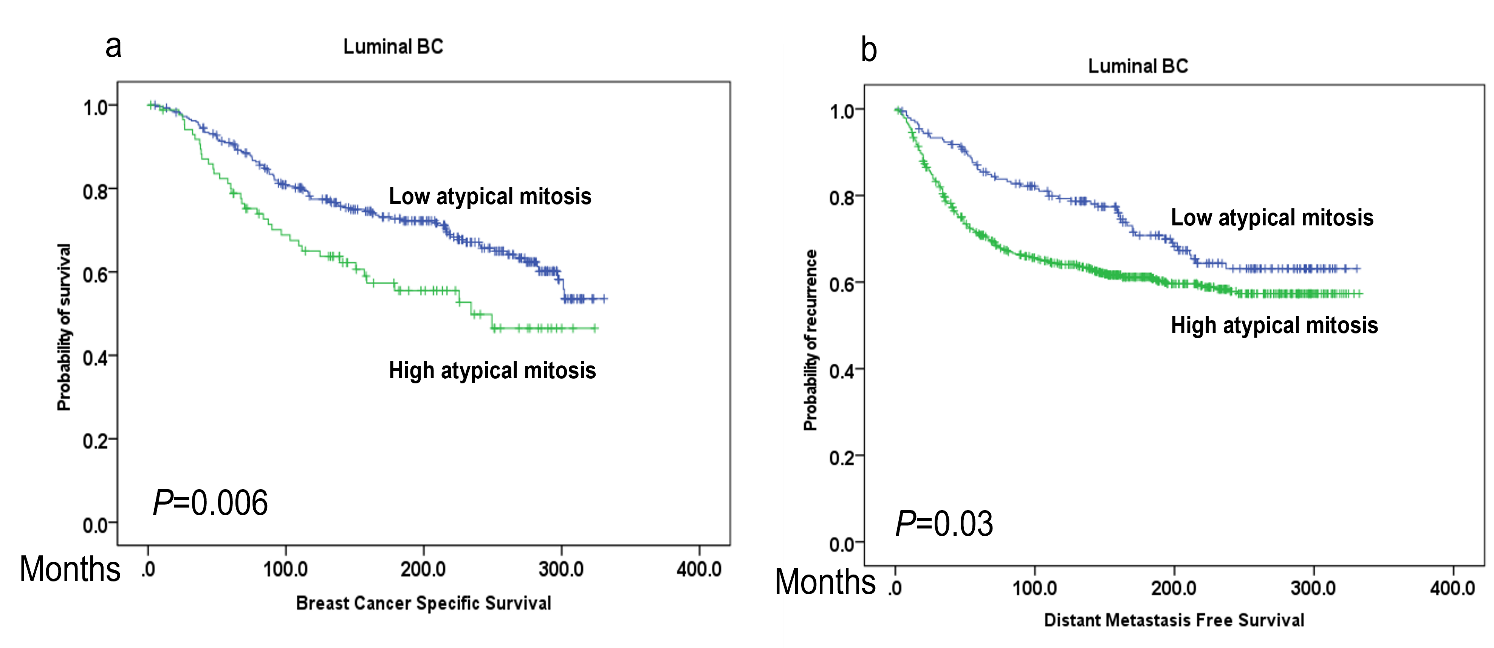


Supplementary figure (2) Kaplan–Meier plots showing (a) associations of BCSSand (b) DMFS with high atypical mitosis in luminal BC subtypes.


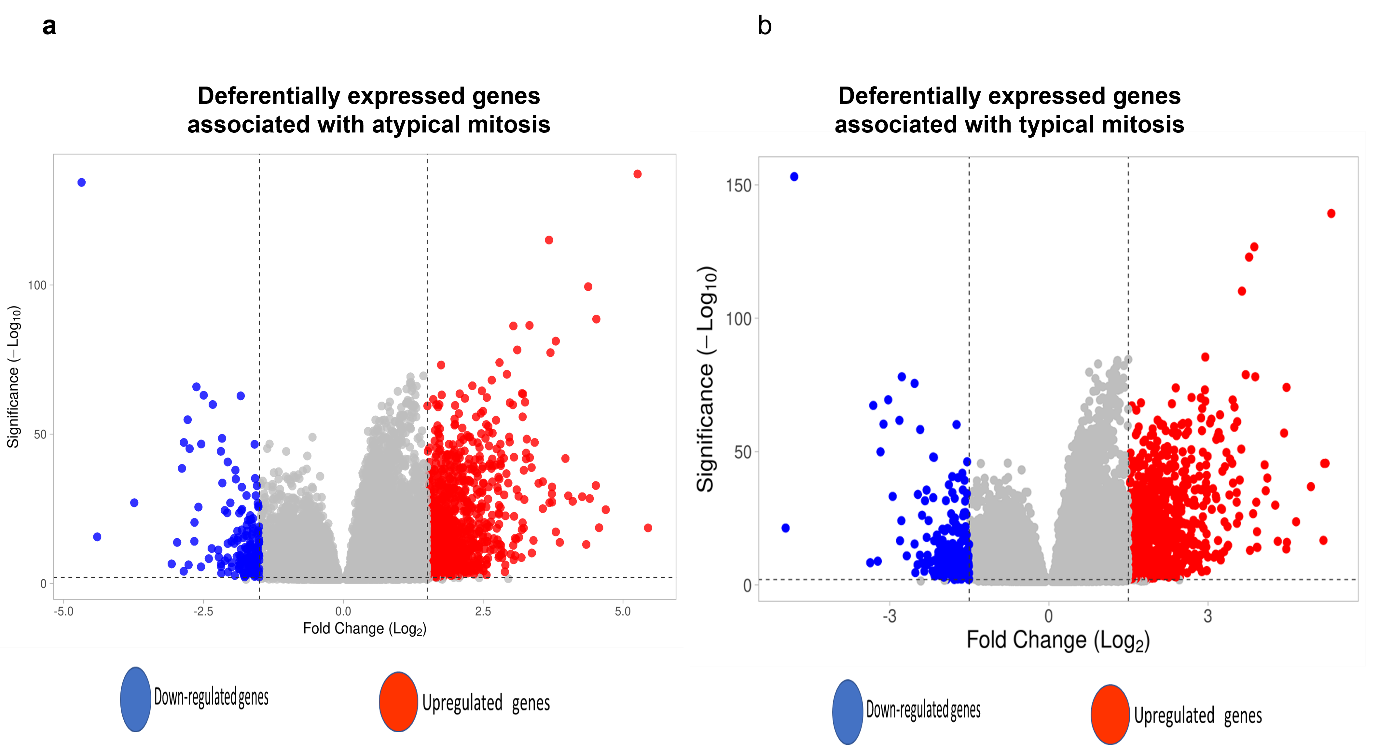


**Supplementary Figure 3:** (a) Volcano plot obtained from differentially gene expression (DGE) analysis (fold change (≥±1) combined with adjusted *p*-value (<0.05) comparing the high versus low atypical mitosis in TCGA cohort while (b) Volcano plot compares high versus low typical mitosis in the same cohort.
